# Detection of Crack Initiation and Growth Using Fiber Bragg Grating Sensors Embedded into Metal Structures through Ultrasonic Additive Manufacturing

**DOI:** 10.3390/s19224917

**Published:** 2019-11-12

**Authors:** Sean K. Chilelli, John J. Schomer, Marcelo J. Dapino

**Affiliations:** Department of Mechanical and Aerospace Engineering, The Ohio State University, Columbus, OH 43210, USA; chilelli.1@osu.edu (S.K.C.); schomer.11@osu.edu (J.J.S.)

**Keywords:** ultrasonic additive manufacturing, UAM, fiber bragg grating, FBG, structural health monitoring, SHM, crack detection

## Abstract

Structural health monitoring (SHM) is a rapidly growing field focused on detecting damage in complex systems before catastrophic failure occurs. Advanced sensor technologies are necessary to fully harness SHM in applications involving harsh or remote environments, life-critical systems, mass-production vehicles, robotic systems, and others. Fiber Bragg Grating (FBG) sensors are attractive for in-situ health monitoring due to their resistance to electromagnetic noise, ability to be multiplexed, and accurate real-time operation. Ultrasonic additive manufacturing (UAM) has been demonstrated for solid-state fabrication of 3D structures with embedded FBG sensors. In this paper, UAM-embedded FBG sensors are investigated with a focus on SHM applications. FBG sensors embedded in an aluminum matrix 3 mm from the initiation site are shown to resolve a minimum crack length of 0.286 ± 0.033 mm and track crack growth until near failure. Accurate crack detection is also demonstrated from FBGs placed 6 mm and 9 mm from the crack initiation site. Regular acrylate-coated FBG sensors are shown to repeatably work at temperatures up to 300 ${}^{\circ }$C once embedded with the UAM process.

## 1. Introduction

Structural health monitoring (SHM) provides improved safety and decreased costs through real-time sensor monitoring of engineering systems. Although SHM has been incorporated in a wide range of applications including civil, aerospace, and industrial systems, there is a need to develop more capable system monitoring and diagnostic tools. SHM has the potential to reduce the need for non-destructive inspection (NDI) which typically requires the system to be out of commission while costly, labor intensive testing is performed [1]. When NDI is relied upon, unsafe conditions due to defects arising during normal operation may go undetected until the next inspection. SHM systems could allow continuous and safer operations where defects are detected in real time. Furthermore, research is being conducted on the use of artificial intelligence, including neural networks, to improve the ability of sensors to detect defects in SHM applications [2].

SHM systems should include a minimally-invasive, long-term sensor network that can accurately detect damage in real time [3]. Fiber Bragg Grating (FBG) strain sensors are currently used in structural applications including buildings foundations [4], wind turbines [5], composite cure monitoring [6], bridges [7], concrete infrastructure [1], and full-scale composite structures [8]. Tosi [9] reviewed chirped FBG sensors that can provide local measurement of strain and temperature along the length of the fiber, typically 15–20 mm, with millimeter-level spatial resolution. Mao et al. [10] demonstrated the use of FBGs for identification of corrosion cracking and expansion in reinforced concrete based on Brillouin optical time domain analysis. FBG sensors have also been used for crack size prediction in conjunction with statistical models [11]. FBG sensors consist of a grating etched into an optical fiber that acts as a light transmission filter, which leads to a specific wavelength being reflected towards the signal source. As the fiber is subjected to either thermal or mechanical loading, strain changes within the sensor induce a change in the reflected wavelength [3]. There are several benefits to FBG sensors that make them effective for SHM. FBG sensors are immune to electromagnetic interference, can be multiplexed, are low cost, require no additional wiring, have high strain resolution, resist corrosion, and are lightweight with a minimally invasive geometry [4]. FBG sensors can provide structural loading data by ensuring strong coupling between the structural matrix and the sensor [12]. Although coupling has been achieved in many polymeric applications through direct embedment, metal systems have historically required sensors to be externally attached to the structure since internal embedment tends to involve process temperatures that are damaging to the sensors [13]. Attempts have been made to use metal-based additive manufacturing (AM) to embed FBG sensors. Selective laser melting (SLM) has been used to embed FBG sensors; however, the residual stresses left by this process tend to induce sensor damage along with poor coupling between the sensor and matrix [14,15]. Some investigations have demonstrated better AM embedment through the use of fibers with specialized coatings [16,17,18].

**Figure 1:**
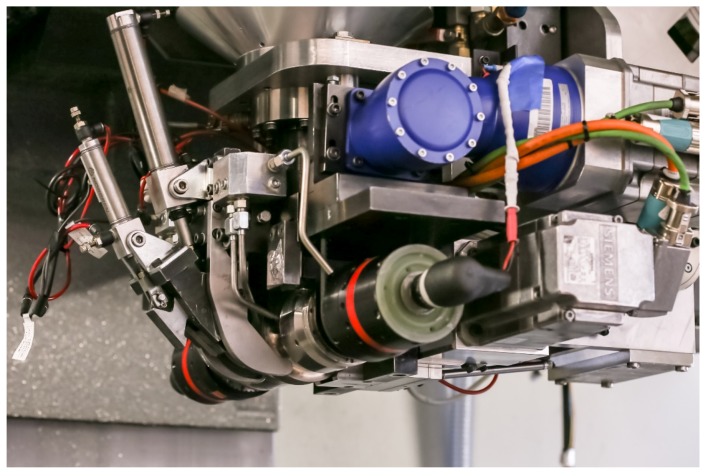
Welding assembly of the 9 kW ultrasonic additive manufacturing welder employed in this study [24].

Unlike most metal forming techniques, UAM is a low-temperature process which allows for sensor embedment without producing mechanical or thermal damage to delicate components. In UAM, successive layers of thin foils are ultrasonically welded on top of each other to build up a metal part [19]. Combined with an incorporated CNC machine, subtractive operations allow for internal features and near-net-shape final parts. UAM builds are accomplished by creating solid-state bonds between layers of metal that are successively welded on top of a metallic baseplate. Metallurgical welding is achieved through direct metal-to-metal contact produced by the simultaneous application of lateral ultrasonic vibrations and mechanical pressure. The combination of high shear strain, normal pressure, and localized temperature increase has the effect of collapsing asperities, scrubbing away the oxide layer, and promoting atomic diffusion from one material to another [20]. The UAM system used in this study is the Fabrisonic SonicLayer 4000, whose welding assembly is shown in Figure 1. UAM can be used to join a variety of similar and dissimilar materials including aluminum alloys [20], steels [21], titanium [22], and carbon fiber composites [23]. Commercial FBG sensors embedded into metal through UAM have been shown to accurately track internal strain, demonstrating the potential of this approach for SHM applications [24,25,26,27]. Plastic flow around UAM-embedded fibers has been previously demonstrated, which leads to strong coupling between the fibers and metal matrix [28]. The main process parameters in UAM are vibration amplitude, down force, and weld speed [20].

In this study, we investigate the use of UAM-embedded FBG sensors in SHM prognostic applications. First, FBG sensors are embedded into compact tension (CT) specimens to determine their effectiveness in detecting crack initiation and growth. Second, embedded FBG sensors are thermally tested to investigate the upper temperature limit of the system. In Section 2, the experimental methods for manufacturing samples with embedded FBG sensors are presented along with the testing procedures. The results for both crack detection and elevated-temperature testing are presented in Section 3. Conclusions are discussed in Section 4.

## 2. Experimental Methods

### 2.1. Sample Fabrication

Test specimens were built using aluminum 6061 due to the well-documented machine parameters available for this alloy [29], though the approach is applicable to other metals. The baseplate was of the T6 condition and the 0.154 mm thick foil layers were of the H18 condition. In this investigation, UAM parameters used for sample fabrication are a downward force of 5000 N, vibration amplitude of 32 μm, and weld speed of 84.6 mm/s.

The general coupon fabrication process is as follows. First, one layer of tape was welded onto the baseplate using UAM. Next, a 0.254 mm by 0.254 mm deep channel was cut using a ball end mill where the fiber would later be embedded. The channels help to avoid cross-sectional loading and sensor deformation during welding [25]. Standard acrylate-coated FBG strain sensors were used as supplied by Moog Inc. along with the wavelength interrogator (Insensys OEM 1030) and analysis software. The FBGs used have a wavelength operating range of 1545 nm to 1555 nm, which corresponds to ±4000 μϵ within the fiber when the nominal wavelength is 1550 nm. The sensors were placed into the channels with the remaining fiber exiting the sample and additional layers were UAM-welded on top to fully encapsulate the FBG sensors at the center of the coupon geometry. After the sensor encapsulation, CNC milling operations were used to build the final sample geometry with the FBG sensor embedded halfway through the sample. The specific geometries created are outlined in the sections below as they vary for different tests. The embedded FBG sensors were examined for changes in their birefringence and polarization response, as it had been documented that the existence of birefringence-induced noise is indicative of an undersized channel and possibly poor bond quality [25]. When time synchronization with other data inputs was necessary, a custom-built analog-to-digital converter was used for converting the serial wavelength output of the interrogator into a voltage that was readable by the data acquisition system.

### 2.2. Crack Propagation

**Figure 2:**
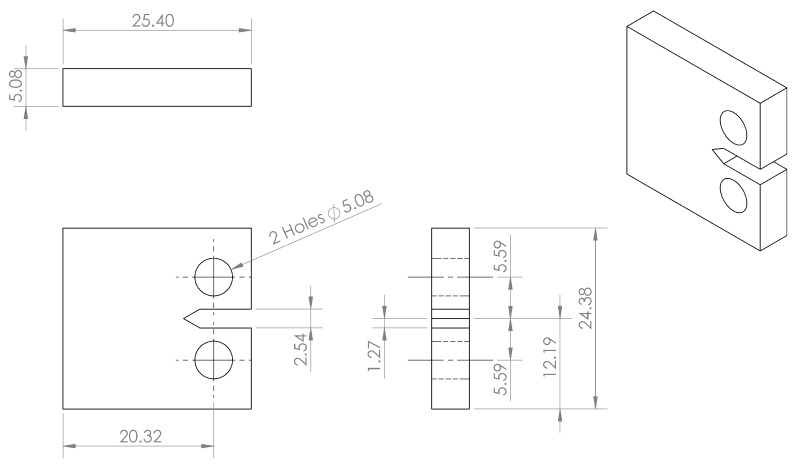
Compact tension (CT) specimen geometry used for the crack detection study. All dimensions are in mm. The FBG sensors were embedded perpendicular to the notch tip.

Tests were conducted to investigate the ability of UAM-embedded FBG sensors to detect cracks in samples. The geometry of the coupons and testing procedures are based on ASTM Standard E647: Standard Test Method for Measurement of Fatigue Crack Growth Rates [30]. This test involves the cyclic tensile testing of notched compact tension (CT) specimens to induce crack growth. A clevis pin system was designed to be held in the load frame and attached to the CT specimen. A drawing of the CT specimen used in this testing is shown in Figure 2.

**Figure 3:**
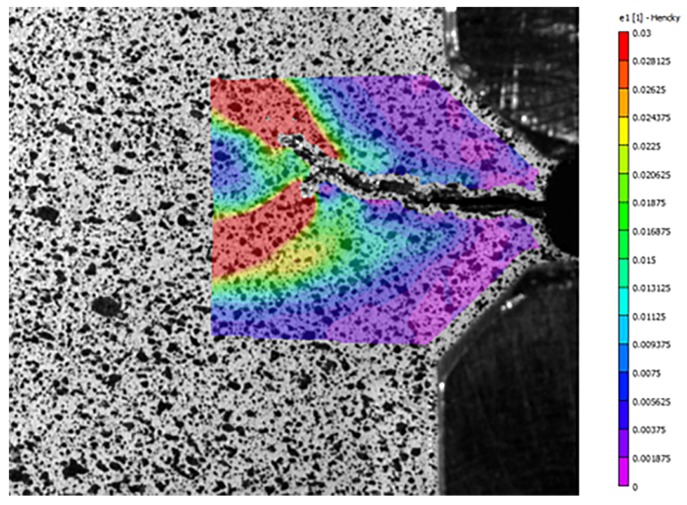
Strain field reported by the DIC system during cyclic loading of a CT specimen.

**Figure 4:**
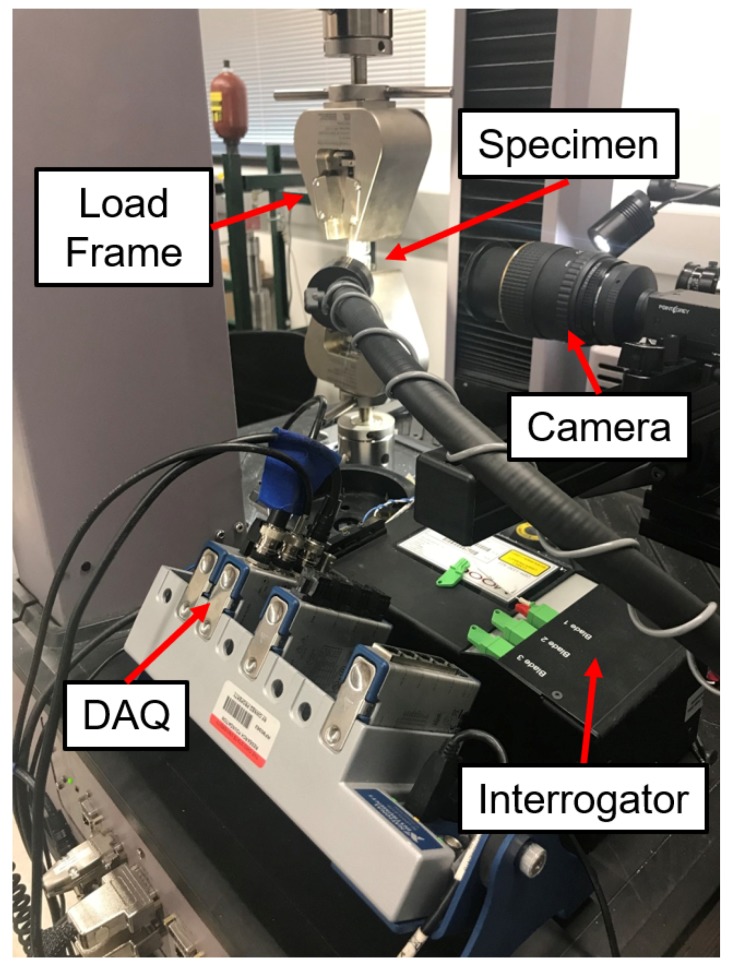
Close-up view of the crack-detection experiment showing a CT coupon installed in the load frame.

Although the purpose of ASTM E647 is for material characterization, we are interested in the strain signal throughout the lifetime of the coupon. For comparison to the strain measurements made by embedded FBG sensors, strain was also measured through digital image correlation (DIC) using a 5-megapixel camera with a 100 mm lens controlled by Vic-Snap 9 Image Acquisition software from Correlated Solutions. DIC is an image processing technique where a speckle pattern is placed on a sample and images are taken throughout testing. DIC analysis tracks the movement of individual speckles and determines the strain field at each image. An example DIC strain field is shown in Figure 3 which shows a strain field overlaid on a close-up image of a CT specimen just before failure. After a set number of cycles, the load frame triggers the DIC to capture an image by having a minimum tensile load slightly smaller than the other cycles. The vertical strain field reported by the DIC system was averaged over the same region as the Bragg grating and used as a comparison to the FBG signal. Crack growth was measured optically using images taken by the DIC system. The length of each pixel corresponded to 0.0054 mm. The loading profile was created using MTS TW Elite software for use with the MTS Criterion Model 43 load frame and a National Instruments 9215 data acquisition module. The experimental setup is shown in Figure 4. There were two main investigations into crack propagation with CT specimens: crack initiation and strain tracking, and prognostic analysis.

#### 2.2.1. Crack Initiation and Strain Tracking

**Figure 5:**
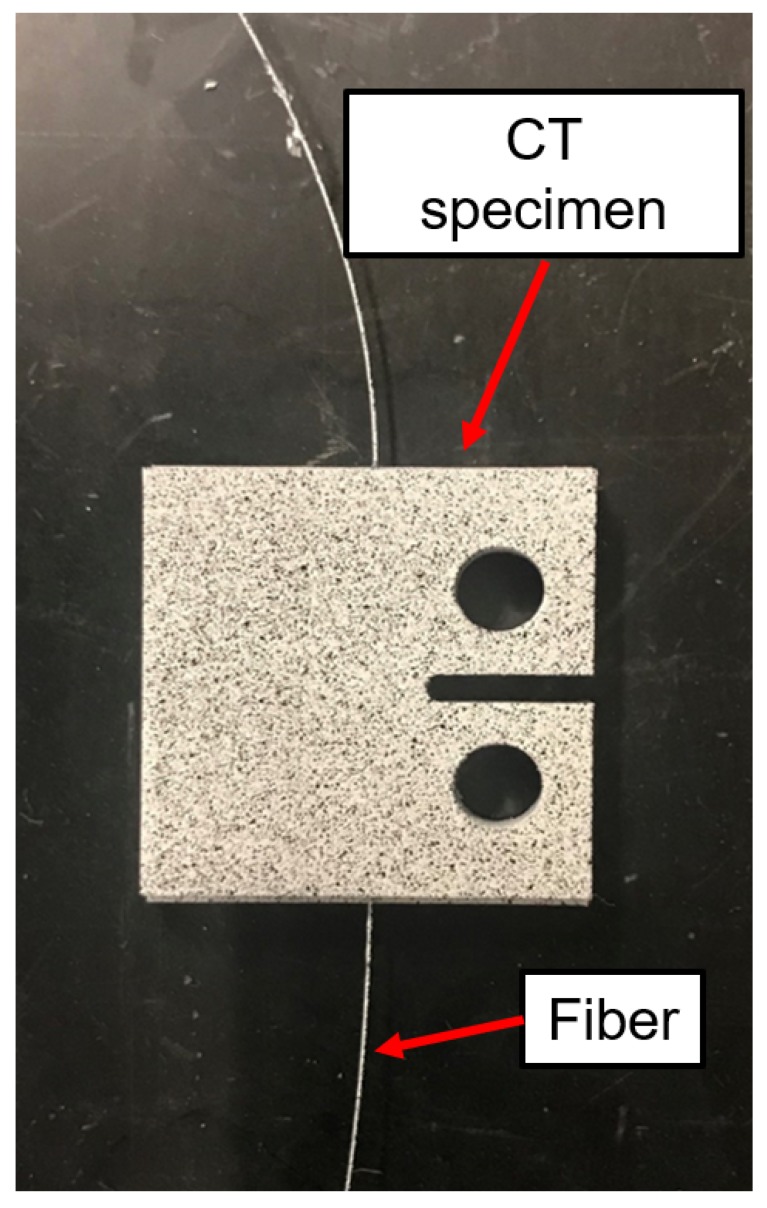
Compact tension (CT) specimen with one embedded FBG sensor built using UAM used in crack initiation and strain tracking. A fine speckle has been applied for DIC tracking. The FBG sensor is located 3 mm from the tip of the notch.

This experiment closely followed ASTM Standard E647. CT specimens with one FBG sensor embedded at a distance of 3 mm from the notch, as in Figure 5, were installed into the load frame and loaded cyclically until the crack reached 1 mm. These pre-cracking cycles consisted of a 0.25 Hz sine wave that alternated from 200 N to 2700 N. After the crack was measured to be greater than 1 mm, the main testing phase began, where the crack was grown until sample failure with the maximum load reduced to 800 N. The DIC system was used to take an image at peak load every 8 cycles during the pre-cracking phase and every 20 cycles during the remainder of the test. The FBG sensor collected data throughout the entire testing procedure. Data from the pre-cracking phase was used to determine the minimum crack size resolved by the FBG sensor. The mean was found for each set of peaks between DIC images. A distribution of the expected mean peak values for each set was found for the region before crack initiation occurred. Assuming this distribution is normal, we define the first set whose mean exceeds three standard deviations of the distribution mean to be the earliest that the FBG can detect the initiation of a crack. Data from the main testing phase was used to determine the ability of the embedded FBG sensors to track strain throughout the growth of a crack.

#### 2.2.2. Prognostic Analysis

**Figure 6:**
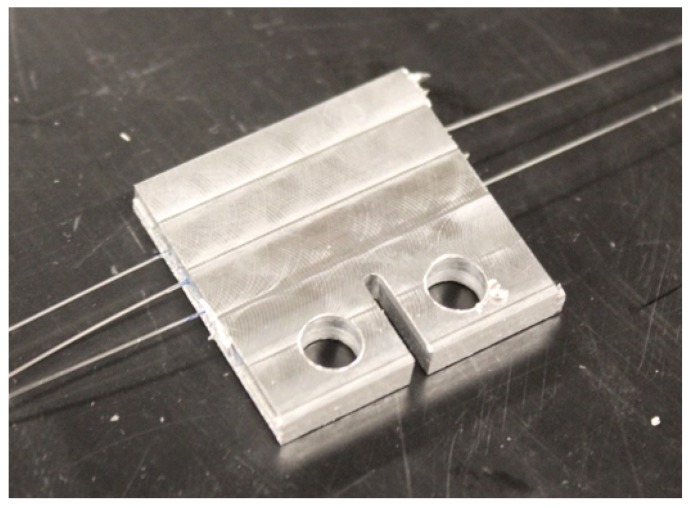
Compact tension (CT) specimen with three embedded FBG sensors built using UAM used in prognostic testing. The sensors are located 3 mm, 6 mm, and 9 mm from the tip of the notch.

In this experiment, a CT specimen was built with three embedded FBG sensors spaced 3 mm, 6 mm, and 9 mm from the notch as pictured in Figure 6. Next, cyclic tensile loading was applied with a 0.25 Hz sine wave. The load profiles alternated between high-amplitude regions to induce crack growth for 200 cycles and low-amplitude regions to test FBG sensing at smaller loads for 30 cycles. In the high-load regions, the tensile loads alternated between 200 N and 2400 N; the tensile loads alternated between 200 N and 800 N in the low-load regions. Once the crack had formed and began propagating, the high-amplitude regions were reduced to 30 cycles. Similar to the previous crack-initiation testing, the wavelength at the peak loads was found before crack initiation had occurred. This was used to estimate the cycle where the expected peak signal exceeds normal bounds in both the high-amplitude and low-amplitude regions for all three embedded FBG sensors.

### 2.3. Elevated-Temperature Testing

After pilot trials revealed accurate strain measurements at elevated temperatures, two tests were conducted to understand the operation of UAM-embedded FBGs at elevated temperatures. In the first test, the set point of the oven temperature increased every 30 min until the FBG failed to produce a signal. Set points used were 50, 75, 100, 150, 200, 250, 300, 350, 400, and 450 ${}^{\circ }$C. In the second test, a coupon was thermally cycled at increasing temperatures. The sample was placed in a cool oven and heated to a set temperature, then the oven was turned off until the temperature returned to room temperature. This was repeated at increasing temperature set points until the FBG failed to produce a signal. Set points used were 50, 75, 100, 150, 200, 250, 300, and 350 ${}^{\circ }$C. At the conclusion of the test, the sample was again heated to 350 ${}^{\circ }$C to evaluate for permanent damage. Samples were then cross-sectioned and micrographs were taken for optical evaluation.

**Figure 7:**
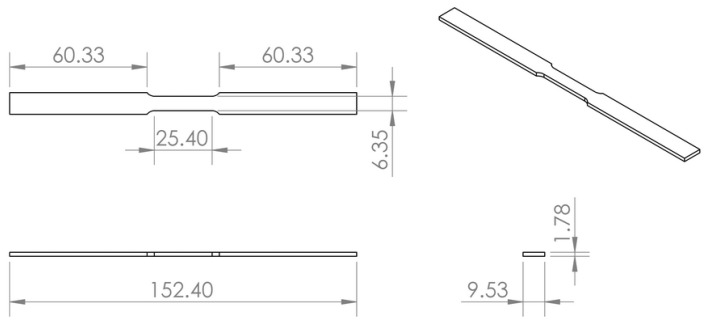
Drawing of coupon used in elevated-temperature testing. All dimensions shown are in mm.

**Figure 8:**
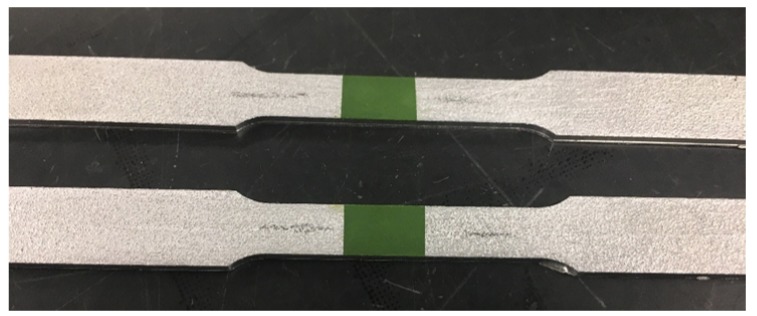
Elevated-temperature coupons built using UAM with a high-temperature strain gauge affixed to the outside of each coupon. FBG fibers are embedded down the length of each coupon.

Coupons were based on ASTM Standard E8: Standard Test Methods for Tension Testing of Metallic Materials, with FBG sensors embedded in the middle of the sample [31]. A drawing of the coupons is shown in Figure 7. A Thermo Scientific Thermolyne furnace was used for testing. The temperature was verified using a K-type thermocouple and the strain was verified using an HPI Inc. HFK-12-125-6-ZCW high-temperature strain gauge that was fixed with epoxy to the coupon as pictured in Figure 8. Data was recorded using a National Instrument 9215 data acquisition module.

## 3. Results and Discussion

### 3.1. Crack Propagation

#### 3.1.1. Crack Initiation and Strain Tracking

**Figure 9:**
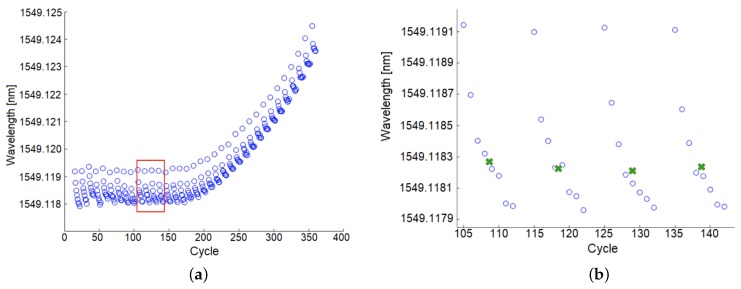
(**a**) Peaks of the FBG strain signal during the pre-cracking phase. The DIC was triggered with a low peak (not shown) every eight cycles. (**b**) Detailed view extracted from the red box. The green x markers indicate the mean of each set.

Pre-cracking data was examined to determine the smallest crack size resolved by UAM-embedded FBGs. As illustrated in Figure 9, which shows the FBG signal peaks of each cycle in the pre-cracking phase, each time the minimum or maximum loads of the load frame were changed, there was a very small, but consistent decrease in the maximum and minimum points over the next few cycles. This effect was repeatable with different FBG sensors and builds, and was also observed in compression (albeit mirrored, with the peaks increasing). Examination of this phenomenon suggests that the decrease in signal peak consistently lasts 20–30 cycles and that the load frame does not exhibit this signal. Additional investigation is underway to understand this phenomenon, though its significance is minimal in practice as the actual strain drop is smaller than 1 microstrain.

A consequence of this phenomenon is that the standard deviation of the normal peak distribution is increased, which reduces the effectiveness of initial crack detection. In the following discussion, the group of peaks after a DIC triggering cycle will be referred to as a set. Instead of using individual peaks before crack initiation, the mean is calculated for each set of 8 cycles, as illustrated in Figure 9b. The means of each set have a much smaller variation and allow for a more precise measurement of crack detection. The mean of the sets before the crack caused any deviation in the digital-to-analog converter signal are used to define a normal distribution with an overall mean of 1549.1181 nm and a standard deviation of 0.0097 nm. We can define the first set that exceeds three standard deviations from this normal mean to be the earliest detection of a crack. The first set whose mean exceeds this range is during cycles 195 to 205, corresponding to a crack length of 0.332 ± 0.046 mm. This analysis is repeated for two additional samples as presented Table 1.

This result illustrates the potential of embedded FBG sensors to detect early crack formation. Comparing this result to other Non-Destructive Investigation (NDI) techniques used in the aerospace industry, shown in Table 2, the UAM-embedded FBG sensors perform about an order of magnitude better than other methods.

It is noted that literature values are the minimum crack detected 90% of the time using the NDI techniques on large parts with unknown cracks. For FBG sensors to be a viable alternative in industry applications, understanding of likely stress concentrations and crack initiation sites is needed in order to optimize FBG sensor location. The tests presented here were carried out with FBG sensors located 3 mm from the crack initiation site. Further investigation of the effects of distance are necessary for informing FBG placement into actual parts. There may be a trade-off between the area of influence of an FBG and the minimum crack size it can detect.

**Figure 10:**
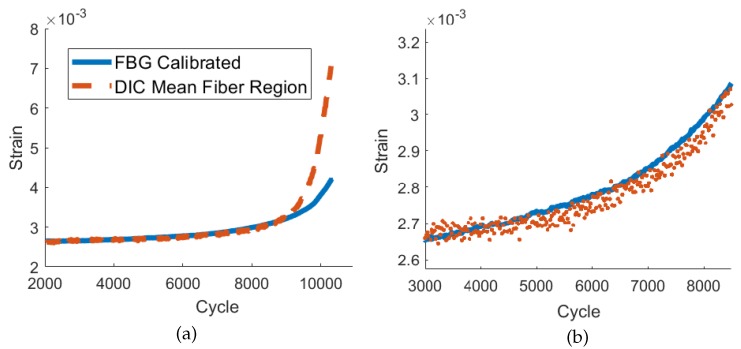
Vertical strain measurements from an FBG sensor UAM-embedded into a CT specimen and average strain from DIC in the region of the FBG; (**a**) from the end of the pre-cracking phase (∼cycle 2000) to sample failure (∼cycle 10500); (**b**) detailed view of (a) from approximately cycle 3000 to 8500.

**Figure 11:**
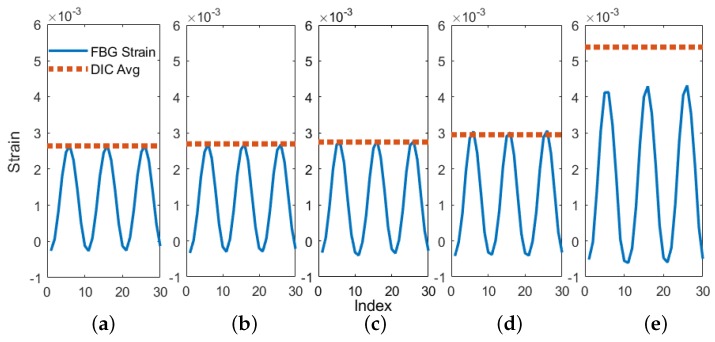
Comparison of the FBG strain signal and the average peak strain from DIC in the region of the FBG after (**a**) 2000, (**b**) 4000, (**c**) 6000, (**d**) 8000, and (**e**) 10,000 cycles.

**Figure 12:**
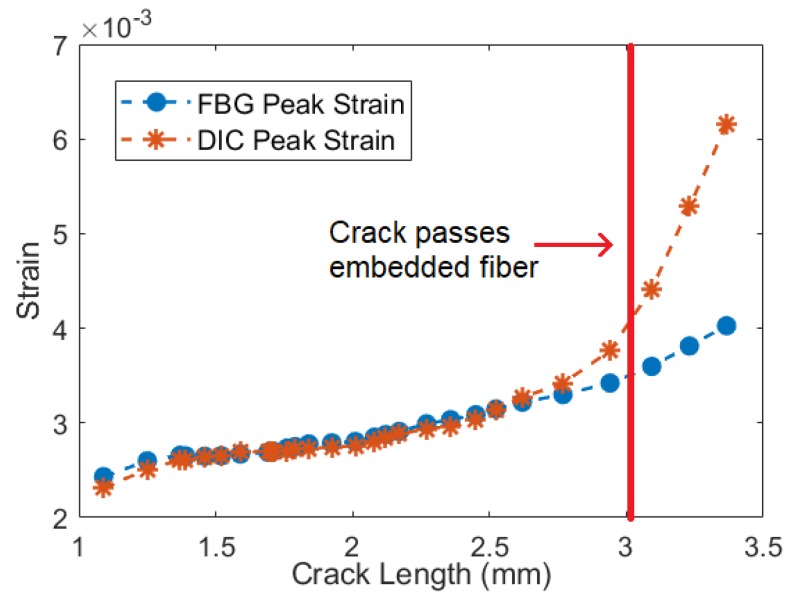
FBG strain signal and average peak strain from DIC in the region of the FBG as the crack length increases.

The results from the main phase of testing provide strong evidence for the ability of UAM-embedded FBG sensors to accurately track strain as a crack propagates toward the sensor. Figure 10, Figure 11 and Figure 12 demonstrate accurate strain tracking for most of the sample’s lifetime. At approximately 9500 cycles, the crack has grown to over 3 mm long and consequently has passed the fiber. At this point the FBG strain begins to deviate from the DIC-measured average strain as the fiber begins to slip within the channel. Prior to slipping, the FBG signal remains extremely accurate as illustrated in Figure 11. In the first 6000 cycles, a ten-cycle moving average of the peak strain of the FBG stays within 1% of the DIC strain, and remains within 5% after 8000 cycles. UAM-embedded FBG sensors are therefore a promising candidate for crack growth tracking in SHM applications.

#### 3.1.2. Prognostic Analysis

**Figure 13:**
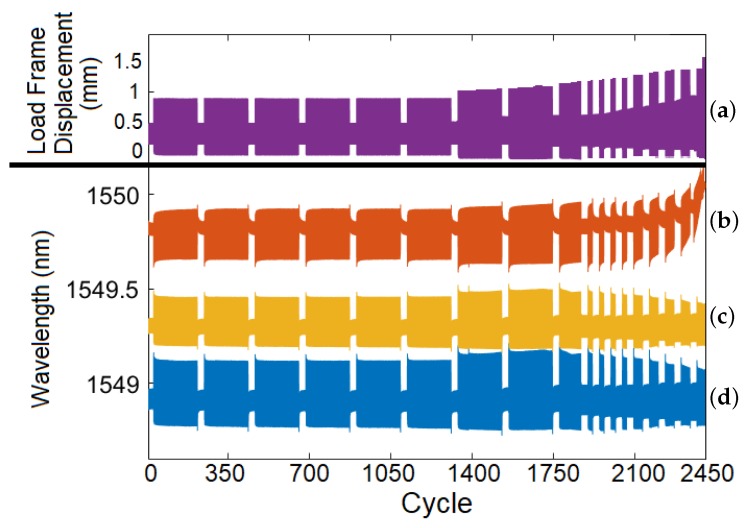
(**a**) Load frame displacement and FBG signals from sensors embedded (**b**) 9 mm, (**c**) 6 mm, and (**d**) 3 mm from the notch over the entire prognostic analysis test.

**Figure 14:**
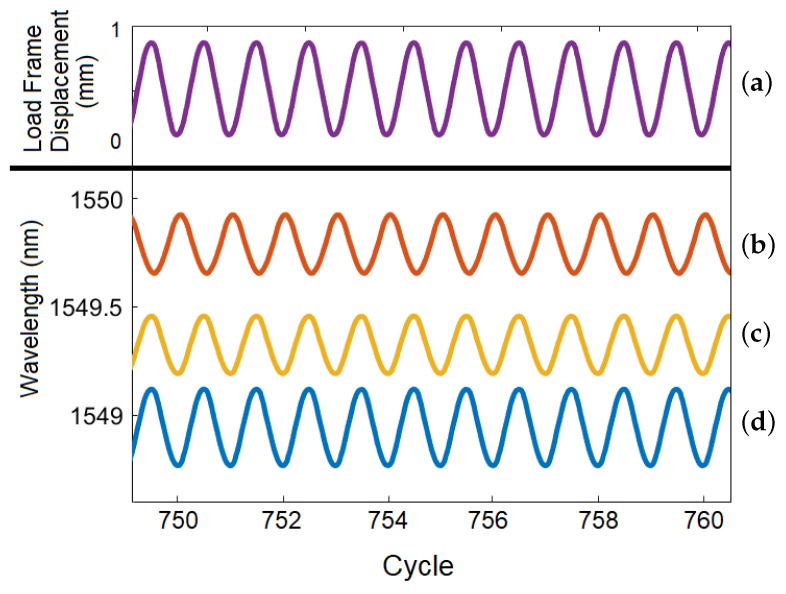
Detailed view extracted from Figure 13 between cycles 749 and 761. (**a**) Load frame displacement and FBG signals from sensors embedded (**b**) 9 mm, (**c**) 6 mm, and (**d**) 3 mm from the notch. Note the alternating crack-growth region (high wavelength amplitude) and low-amplitude region (low wavelength amplitude). The number of cycles of the crack-growth phase was reduced toward the end of the test to ensure that enough data was obtained as the crack grew.

The prognostic analysis investigation used a CT specimen with three FBG sensors embedded at 3 mm, 6 mm, and 9 mm from the notch as shown in Figure 6. There were two main alternating phases of the load profile. In the high-amplitude, crack-growth phase, the investigation focused on determining the minimum detectable crack length. In the low-amplitude phase, the signal with no crack was compared to the case where a slowly-growing crack was present. The signals from all three embedded FBG sensors were analyzed to compare how distance from the initiation site affects the results. The test results are shown in Figure 13 and Figure 14. The 3 mm and 6 mm FBG signals are out of phase with respect to the 9 mm FBG signal, as stress builds up unevenly in the CT specimen; this causes the side opposite the notch to be compressed when the load frame loads the coupon in tension [33].

**Figure 15:**
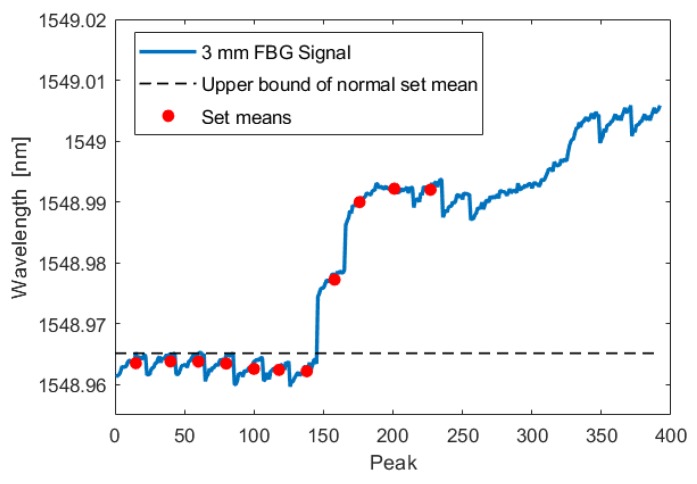
Crack detection analysis during low-amplitude cyclic loading for the FBG embedded 3 mm from the notch tip. For this and subsequent images, the dashed line represents three standard deviations above the normal set mean.

**Figure 16:**
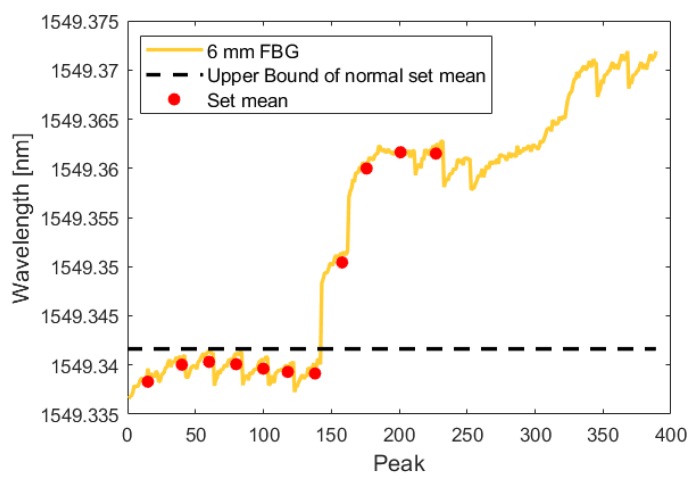
Crack detection analysis during low-amplitude cyclic loading for the FBG embedded 6 mm from the notch tip.

**Figure 17:**
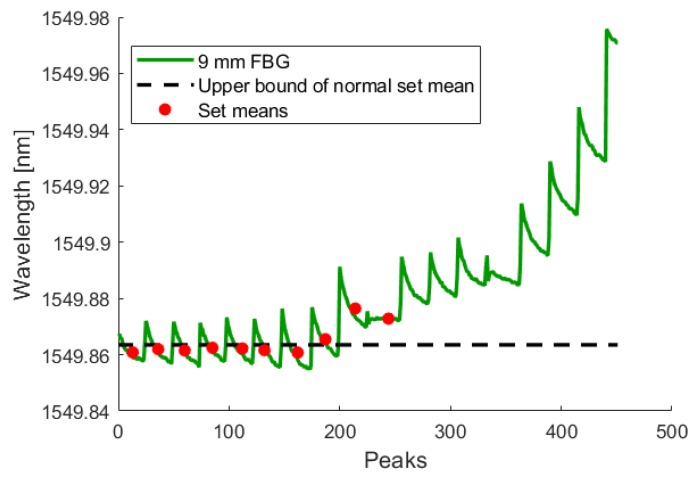
Crack detection analysis during low-amplitude cyclic loading for the FBG embedded 9 mm from the notch tip.

**Figure 18:**
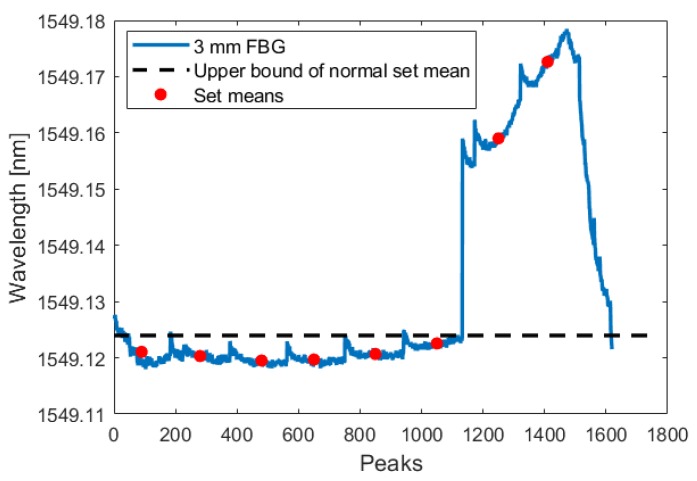
Crack detection analysis during high-amplitude cyclic loading for the FBG embedded 3 mm from the notch tip.

**Figure 19:**
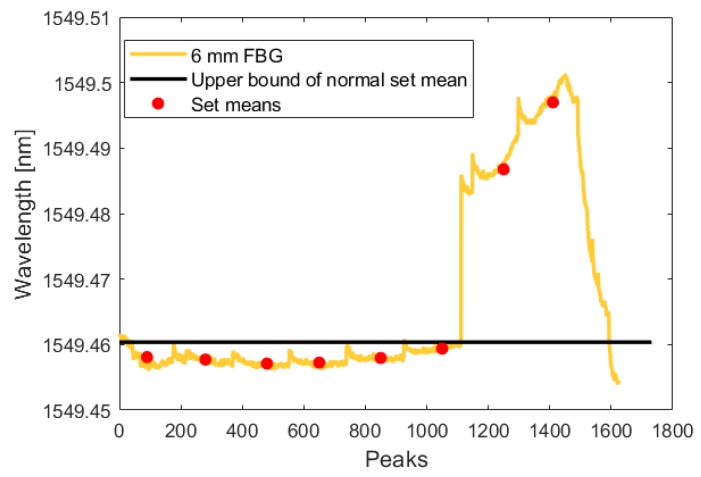
Crack detection analysis during high-amplitude cyclic loading for the FBG embedded 6 mm from the notch tip.

**Figure 20:**
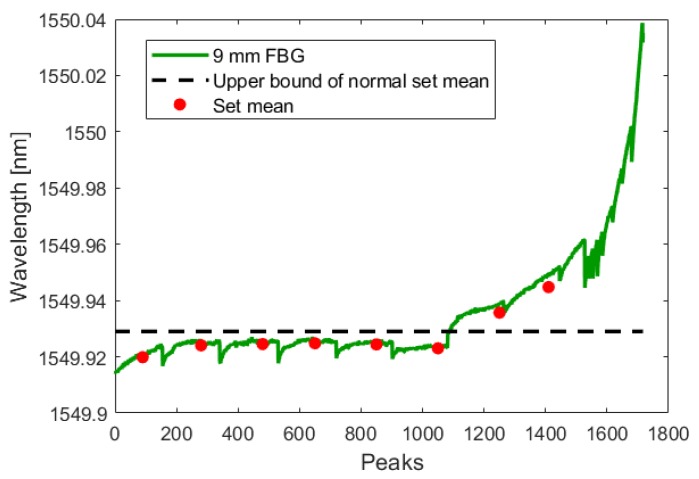
Crack detection analysis during high-amplitude cyclic loading for the FBG embedded 9 mm from the notch tip.

During the test, the crack initiated and grew in a single cycle to a length of 0.350 mm. Using the analysis technique described in Section 3.1.1, this increased crack length was detected by all three fibers in both the crack-growth phase and the low-amplitude phase. The following figures show the FBG signal as a solid line and the calculated upper bound of set means with no crack as a dotted line. The red circles indicate the mean of each set. When the red circle crosses above the dotted line, the FBG detects a crack. As illustrated by Figure 15, Figure 16, Figure 17, Figure 18, Figure 19 and Figure 20, all embedded FBGs successfully detect the crack after the seventh high-amplitude set when the crack reaches a length of 0.350 mm.

Although the signal change was less clear for FBG signals farther away from the notch, the fact that all three FBG sensors detect the crack growth illustrates the potential robustness of UAM-embedded FBG sensors in detecting crack initiation. Furthermore, the detection of the crack in the low-amplitude phase illustrates the ability of FBG sensors to detect minute defects even at normal operating conditions. Additional testing with more FBG sensors at more locations could provide insight into the relationship between crack detection and embedded sensor locations.

### 3.2. Elevated-Temperature Testing

**Figure 21:**
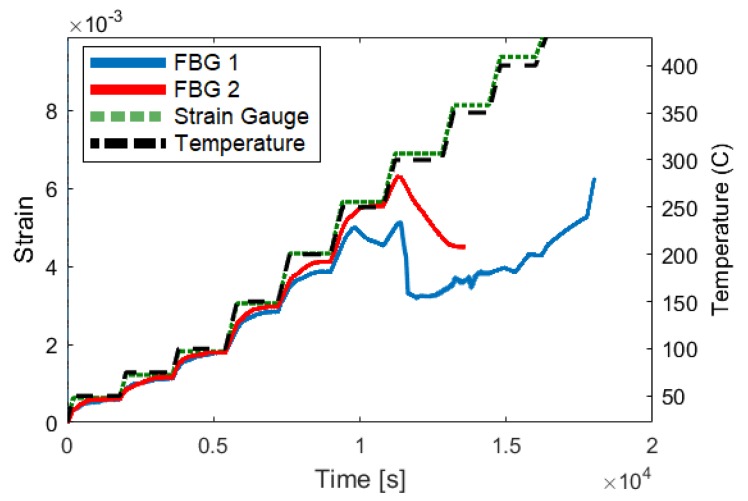
Elevated-temperature testing of two UAM-embedded FBG sensors. The strain measurements of two coupons with embedded FBG sensors and strain gauges is shown using the left scale. The temperature trace (black line) follows the scale on the right.

Initial pilot investigations show that UAM-embedded FBG sensors may be able to accurately detect strain at temperatures higher than typical acrylate-coated FBG sensors. To investigate this further, the test shown in Figure 21 was conducted. This test illustrates that the FBG signal is able to accurately track the increasing temperature set points up to temperatures between 200 and 275 ${}^{\circ }$C.

**Figure 22:**
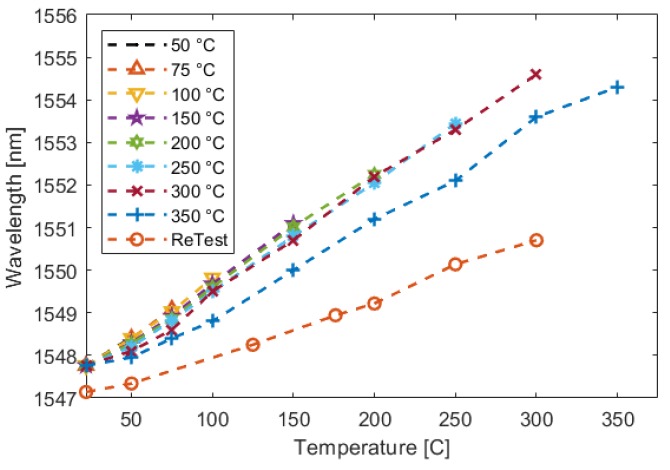
Elevated-temperature cyclic testing of an UAM-embedded FBG sensor. The FBG signal was measured during a cyclic temperature test with increasing oven set points. Once noticeable deviation occurred at 350 ${}^{\circ }$C, the test was performed one more time to confirm that permanent damage had occurred.

Figure 22 shows a second elevated-temperature test where a sample exhibits a repeatable signal up to 300 ${}^{\circ }$C. At the 350 ${}^{\circ }$C set point, there is a clear deviation from the previous signal. Additional testing confirmed that the fiber was permanently degraded.

**Figure 23:**
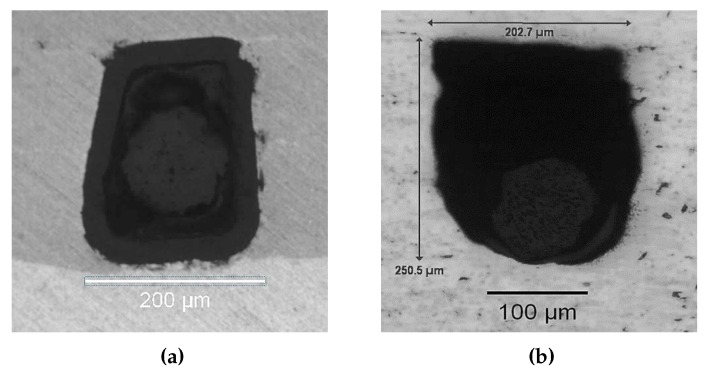
(**a**) Cross-section of an UAM-embedded FBG that has not undergone thermal loading and (**b**) cross-section of the UAM-embedded FBG that underwent cyclic thermal testing.

Samples were then cross-sectioned and examined through optical microscopy to identify any visible changes in structure. As presented in Figure 23, the control sample shows the clear and intact structures of the fiber: outer coating, inner coating, and cladding (the core is not visible unless illuminated from behind). However, in the sample that was subjected to a temperature of 350 ${}^{\circ }$C, the outer and inner coatings appear mostly gone and the cladding is no longer centered in the channel. This result was consistent across samples from both tests.

## 4. Concluding Remarks

Structural Health Monitoring (SHM) techniques are underused in many industries due to sensor limitations. Fiber Bragg Grating strain sensors that have been embedded into metal through Ultrasonic Additive Manufacturing (UAM) are promising for SHM applications. UAM-embedded FBG sensors were shown to detect and track crack growth through the life of a CT specimen. Embedded FBG sensors were able to closely monitor crack growth until the crack passed the embedded fiber and the fiber began to slip. Embedded FBG sensors can enable early crack detection, with sensors 3 mm from the crack initiation point detecting cracks with a length of 0.286 ± 0.033 mm, an order of magnitude better than traditional NDI techniques. Even at distances of 6 mm and 9 mm, a crack size of 0.350 mm was resolved during both high loads (crack-growth phase) and low loads (normal operating phase). This result demonstrates the potential for FBG sensors to act as prognostic tools during the operation of components. Embedded FBG sensors were also shown to work at elevated temperatures, highlighting the possibility of higher temperature applications up to 300 ${}^{\circ }$C. UAM-embedded FBG sensors have been shown to be an effective tool for structural health monitoring of complex systems.

## Figures and Tables

**Table 1:** Length of crack in CT specimen at earliest detection using an UAM-embedded FBG sensor located 3 mm from the notch. Tolerances are due to crack entering a DIC speckle and consequently being unable to optically determine a precise endpoint.

Sample	Crack Size [mm]
1	0.332 ± 0.046
2	0.234 ± 0.054
3	0.291
Average	0.286 ± 0.033

**Table 2:** Comparison of minimum detectable crack size between UAM-embedded FBG sensors and common aerospace NDI techniques [32].

Technique	Crack Size [mm]
UAM FBG	0.286 ± 0.033
Eddy Current	2.54
Penetrant	3.81
Magnetic Particle	6.35

## Abbreviations

The following abbreviations are used in this manuscript:

UAM	Ultrasonic Additive Manufacturing
FBG	Fiber Bragg Grating
SHM	Structural Health Monitoring
CT	Compact Tension
DIC	Digital Image Correlation
NDI	Non-Destructive Inspection
